# Antipsychotic drug—aripiprazole against schizophrenia, its therapeutic and metabolic effects associated with gene polymorphisms

**DOI:** 10.1007/s43440-022-00440-6

**Published:** 2022-12-16

**Authors:** Adriana Stelmach, Katarzyna Guzek, Alicja Rożnowska, Irena Najbar, Anna Sadakierska-Chudy

**Affiliations:** 1 Department of Genetics, Faculty of Medicine and Health Sciences, Andrzej Frycz Modrzewski Krakow University, Gustawa Herlinga-Grudzinskiego 1, 30-705 Krakow, Poland; 2 Centre of Education, Research and Development, Babinski University Hospital, Krakow, Poland

**Keywords:** Antipsychotics, Aripiprazole, CYP450 system, Genetic polymorphism, Pharmacogenetics, Schizophrenia, Treatment response

## Abstract

Second-generation antipsychotics are widely used for the treatment of schizophrenia. Aripiprazole (ARI) is classified as a third-generation antipsychotic drug with a high affinity for dopamine and serotonin receptors. It is considered a dopamine-system stabilizer without severe side effects. In some patients the response to ARI treatment is inadequate and they require an effective augmentation strategy. It has been found that the response to the drug and the risk of adverse metabolic effects can be related to gene polymorphisms. A reduced dose is recommended for CYP2D6 poor metabolizers; moreover, it is postulated that other polymorphisms including *CYP3A4*, *CYP3A5*, *ABCB1*, *DRD2,* and *5-HTRs* genes influence the therapeutic effect of ARI. ARI can increase the levels of prolactin, C-peptide, insulin, and/or cholesterol possibly due to specific genetic variants. It seems that a pharmacogenetic approach can help predict drug response and improve the clinical management of patients with schizophrenia.

## Introduction

Antipsychotics are used to treat mental illnesses such as schizophrenia and other psychosis, as well as bipolar disorder and depression. These medications are divided into three groups: (1) first-generation antipsychotics (FGAs); (2) second-generation antipsychotics (SGAs); and (3) third-generation antipsychotics (TGAs) [1, 2]. The main difference between FGAs, SGAs, and TGAs is their pharmacological target. FGAs, such as haloperidol, chlorpromazine, and thioridazine, act mainly on the dopaminergic system as antagonists for the dopamine type 2 (D2) receptors [3]. They alleviate positive symptoms of schizophrenia; however, D2 blockade often induces numerous side effects, the most prominent are extrapyramidal symptoms (EPS) [3]. In addition, the blockade of D2 receptors causes an increase in prolactin levels that correlates with the dose [4]. SGAs, including risperidone and clozapine, have a higher affinity for serotonin receptors (5-HT) than D2 receptors [5]. Due to 5-HT_2A_/D2 antagonist properties, they are also called dopamine-serotonin antagonists. Moreover, SGAs exhibit an action on muscarinic cholinergic receptors (M3), histamine receptors (H1), and adrenergic receptors (α1 and α2) [6]. Although SGAs are associated with a lower risk of EPS they can cause metabolic effects, such as weight gain, diabetes mellitus, hyperlipidemia, QT modifications, and hyperprolactinemia [7]. TGAs, including aripiprazole (ARI), brexpiprazole, and cariprazine are partial dopamine receptor agonists and also act as antagonists or weak partial agonists on the serotonin receptors [1, 8–10].

ARI, apart from being described as an antipsychotic drug, is also a mood stabilizer. Antipsychotic medications are mainly used for treating schizophrenia; however, they are effective for other psychotic disorders and also other psychiatric disease entities, such as mania, bipolar affective disorder, depression, anxiety disorders, delusional disorders, irritability associated with autism, Tourette's syndrome, disorders associated with problems with impulse control, and behavioral disturbances in dementias or in children and adolescents. Short-acting ARI is also used intramuscularly for rapid tranquillization in acute agitation associated with schizophrenia or bipolar disorder [11, 12]. Detailed information about antipsychotic drug metabolism, mechanism of action, and adverse side effects is shown in Table 1.

**Table 1:** Characteristics of selected antipsychotic drugs

Drug	Metabolism	Mechanism of action	Recommended dose	Adverse side effects	References
First-generation drugs					
Low potency					
Chlorpromazine	Hydroxylation, demethylation catalyzed by cytochrome P450 isozymes CYP2D6, CYP1A2, CYP3A4	D1, D2, D3, D4, 5-HT1, 5-HT-2, H1, α1, α2-adrenergic, M1, M2 antagonist	400 mg/day	Anticholinergic effects, dyslipidemia, extrapyramidal symptoms, neuroleptic malignant syndrome, postural hypotension, prolonged QT interval, sedation, seizures, sexual dysfunction, diabetes mellitus type 2, weight gain, cardiovascular events, pulmonary embolism, venous thromboembolism	[26–29] DrugBank Online [https://go.drugbank.com/drugs/ DB00477] Drugs.com [https://www.drugs.com/dosage/chlorpromazine.html]
Thioridazine	S-oxidation, N-dealkylation catalyzed by cytochrome P450	D1, D2, α1 -adrenergic antagonist	200–300 mg/day	Anticholinergic effects, dyslipidemia, extrapyramidal symptoms, neuroleptic malignant syndrome, postural hypotension, very high risk of prolonged QT interval, sedation, seizures, sexual dysfunction, diabetes mellitus type 2, weight gain	[26, 30–33] DrugBank Online [https://go.drugbank.com/drugs/ DB00679] Drugs.com [https://www.drugs.com/dosage/thioridazine.htm]
High potency					
Haloperidol	Reduction, glucuronidation catalyzed by UDP-glucuronosyltransferase Oxidation catalyzed by cytochrome P450 enzymes	D2, 5-HT2A, α1- adrenergic, H1 antagonist	15 mg/day	Parkinsonism, dystonia, akathisia, tardive dyskinesia, dry mouth, constipation, delirium, memory deficits, QT prolongation, prolactin elevation, sexual dysfunction	[1, 11, 29, 32, 34–36] DrugBank Online [https://go.drugbank.com/drugs/DB00502]
Pimozide	N-dealkylation catalyzed by cytochrome P450 isoenzymes CYP3A and CYP1A2	D2, D3 antagonist calmodulin inhibitor	2–4 mg/day	Extrapyramidal effects, prolactin elevation, sedation, anticholinergic effects	[37–40] DrugBank Online [https://go.drugbank.com/drugs/ DB01100]
Fluphenazine	S-oxidation catalyzed by cytochrome P450 isoenzyme CYP2D6	D1, D2 antagonist calmodulin inhibitor	20 mg/day	Very often EPS and hyperprolactinemia, dyslipidemia, neuroleptic malignant syndrome, postural hypotension, prolonged QT interval, sedation, seizures, sexual dysfunction, diabetes mellitus type 2, weight gain	[41–43] DrugBank Online [https://go.drugbank.com/drugs] Drugs.com [https://www.drugs.com/mtm/fluphenazine.html]
Second-generation drugs					
Olanzapine	N-glucuronidation catalyzed by UGT1A4 and UGT2B10 Oxidation reactions catalyzed by cytochrome P450 enzymes N-oxidation reactions catalyzed by the flavin monooxygenase 3	D2, D3, D4, 5-HT2A, 5-HT2B, 5-HT2C, 5-HT3, 5-HT6, H1, α1-adrenergic, M1-M5 antagonist	20 mg/day	Weight gain, insulin resistance, hyperglycemia, high triglycerides, VLDL cholesterol and triglyceride level, constipation, dizziness, personality disorder, akathisia, postural hypotension, sedation, headache, increased appetite, fatigue, dry mouth, leukopenia, thrombocytopenia, bradykinesia, parkinsonism dystonia	[9, 44–49] DrugBank Online [https://go.drugbank.com/drugs/DB00334]
Clozapine	N-demethylation N-oxidation glucuronidation	D2, D1, D3, D4, 5-HT2A, H1, H4, M1-M5 antagonist	300–450 mg/day	Anticholinergic effects, dyslipidemia, neuroleptic malignant syndrome, postural hypotension, prolonged QT interval, sedation, seizures, sexual dysfunction, diabetes mellitus type 2, weight gain, neutropenia, agranulocytosis, tachycardia, myocarditis, cardiomyopathy, gastrointestinal motility disorders	[41, 49–54] DrugBank Online [https://go.drugbank.com/drugs/ DB00363]
Risperidone	Hydroxylation, N-dealkylation catalyzed by cytochrome P450 CYP2D6 isozyme	5-HT2A, 5-HT2C, 5-HT1A, 5-HT2D, 5-HT7, D2, α1-adrenergic, α2-adrenergic, H1 antagonist	2–8 mg/day	Dyslipidemia, extrapyramidal syndrome, hyperprolactinemia, neuroleptic malignant syndrome, postural hypotension, prolonged QT interval, sedation, seizures, sexual dysfunction, diabetes mellitus type 2, weight gain	[9, 26, 55, 56] DrugBank Online [https://go.drugbank.com/drugs/DB00734] Drug.com [https://www.drugs.com/dosage/risperidone.html]
Quetiapine	Sulfoxidation and oxidation catalyzed by cytochrome P450 3A4, CYP2D6 isoenzymes	5HT2A, D2, H1, M1–M5 antagonist	300–600 mg/day	Anticholinergic effects, dyslipidemia, neuroleptic malignant syndrome, postural hypotension, prolonged QT interval, sedation, seizures, sexual dysfunction, diabetes mellitus type 2, weight gain	[11, 26, 57–60] DrugBank Online [https://go.drugbank.com/drugs/DB01224]
Ziprasidone	Oxidation, Oxidative N- dealkylation, glucuronidation, Oxidative N-dealkylation, methylation, S-oxidation, dearylation, hydration catalyzed by aldehyde oxidase and by CYP3A4	D2, 5-HT2A, H1, M1–M5 antagonist	40–80 mg/day	Extrapyramidal symptoms, Hyperprolactinemia, neuroleptic malignant syndrome, postural hypotension, prolonged QT interval, sedation, seizures, sexual dysfunction, diabetes mellitus type 2	[26, 59, 61–63] DrugBank Online [https://go.drugbank.com/drugs/ DB00246]
Third-generation drugs					
Aripiprazole	dehydrogenation, hydroxylation, N-dealkylation catalyzed by cytochrome P450 enzymes	D2, D3, 5-HT1A, 5-HT2A, 5-HT2C and 5-HT7 partial agonist 5-HT2B agonist α1A adrenergic, H1 and 5-HT6 antagonist α2 adrenergic, M1 low affinity	10–30 mg/day	Extrapyramidal effects, headache, agitation, insomnia, anxiety, nausea and vomiting, akathisia, light-headedness, constipation	[9, 64–66] DrugBank Online [https://go.drugbank.com/drugs/DB01238] Drugs.com [https://www.drugs.com/dosage/aripiprazole.html]
Brexpiprazole	S-oxidation catalyzed by CYP3A4 and CYP2D6	5-HT1A, D2 agonist, 5HT2A, α2C, α1B-adrenergic antagonist	2–4 mg/day	Weight gain, extrapyramidal symptoms, prolactin elevation, sedation, anticholinergic effects, dyslipidemia	[37, 67–69] DrugBank Online [https://go.drugbank.com/drugs/DB09128]
Cariprazine	CYP3A4, and less extent by CYP2D6	Mostly target D2 and 5-HT2A receptors, also partial 5-HT1A agonist, 5-HT2B and 5-HT2A antagonist, histamine H1 receptors antagonist	1.5–6 mg/day	Extrapyramidal symptoms, akathisia	[67, 70] DrugBank Online [https://go.drugbank.com/drugs/DB06016] Drugs.com https://www.drugs.com/mtm/cariprazine.html

Schizophrenia is a chronic, serious mental illness that affects about 1% of the world’s population. The pathomechanism of this disease is complex and still not sufficiently understood. It is plausible that genetic and environmental factors as well as other causes, such as brain chemistry, substance use, and autoimmune diseases or inflammation play a role in the risk of developing schizophrenia. The disease begins at a young age (usually before the age of 18) and causes different symptoms: (1) positive (e.g., hallucinations, delusions, disorganized behavior); (2) negative (e.g., social withdrawal, apathy, lack of energy, anhedonia, flattened affect); and (3) cognitive (e.g., memory and learning impairments or attention deficiencies) [9, 11]. Several SGAs are currently available for the treatment of schizophrenia including clozapine, olanzapine, and risperidone. However, these drugs can cause metabolic adverse effects, in turn, a TGA drug, ARI, appeared to have general advantages regarding side effects [13]. ARI is generally well-tolerated, it has a low propensity for EPS and causes lower incidences of excessive weight gain, glucose dysregulation, hypercholesterolemia, and hyperprolactinemia [14, 15]. The latter seems to be very important for women because high prolactin levels increase the risk of developing breast cancer [16, 17]. A study carried out on female patients with schizophrenia showed that long-term exposure to ARI (prolactin-sparing antipsychotic) was not associated with an increased risk of breast cancer [18]. Moreover, a clinically significant property is that ARI is not associated with impaired glucose tolerance, which is particularly important in pregnant women [19]. It is well known that the disturbance in glucose during pregnancy can increase the risk of gestational mellitus diabetes [20]. Several studies have shown that ARI, both oral form, and long-acting injection, was not associated with an increased risk of major congenital defects and neurological malformations [21–23]. Surprisingly, it was established that ARI (10–20 mg/day) has the potency to normalize significantly elevated levels of serum prolactin caused by antipsychotic-induced prolactinemia [24, 25]. Although the severity of ARI side effects, including EPS and metabolic syndromes, is less frequent than with other antipsychotics, some patients have experienced adverse drug effects.

The purpose of this review is to highlight the role of various genetic polymorphisms in the pharmacokinetics and pharmacodynamics of ARI. This review also offers a brief discussion of the relationship between genetic variants and metabolic side effects. Understanding the role of polymorphisms in the efficacy and safety of aripiprazole therapy may in the future result in the translation of pharmacogenomic knowledge into clinical practice.

## Mechanism of action of ARI

ARI was approved in the USA in 2002, in Europe in 2004, and in Japan in 2006 for indication of schizophrenia [71]. It acts as a partial D2 dopamine and serotonin 5-HT_1A_ receptors agonist as well as a serotonin 5-HT_2A_ receptor antagonist. Furthermore, the affinity of ARI for other crucial nervous system receptors has been demonstrated (Table 2).

**Table 2:** Aripiprazole affinity for human receptors

Types	Affinity	Affinity	Ki (nmol/L)	Biological action	Pharmacological action
Dopamine receptors					
D2	+ + + +	Very high	0.34^#^	Partial agonist	Yes
D3	+ + + +	Very high	0.8^#^		Unknown
D4	+	Moderate	44^#^		Unknown
Serotonin receptors					
5-HT2B	+ + + +	Very high	0.36^##^	Inverse agonist	Unknown
5-HT1A	+ + +	High	1.7^#^	Partial agonist	Unknown
5-HT2A	+ +	Moderate	3.4^#^	Antagonist/partial agonist	Yes
5-HT2C	+	Limited	15^#^	Partial agonist	Unknown
5-HT7	+ + +	High	39^#^ (10^##^)	Partial agonist	Unknown
5-HT1D	+ +	Moderate	68^##^	Antagonist	Unknown
5-HT6	+	Limited	570^##^	Antagonist	Unknown
5-HT1B	+	Limited	830^##^	Antagonist	Unknown
Noradrenaline receptors					
α1A	+ +	Moderate	57^#^ (26^##^)	Antagonist	No
α1B	+ +	Moderate	35^##^	Antagonist	No
α2C	+ +	Moderate	38^##^	Antagonist	No
α2A	+ +	Moderate	74^##^	Antagonist	No
α2B	+	Limited	103^##^	Antagonist	No
Histamine receptors					
H1	+ +	Moderate	61^#^ (25^##^)	Antagonist	No

***Ki*** the binding affinities were assessed in vitro

^#^Abilify Maintena prescribing information [ABILIFY MAINTENA—Accessdata.fda.gov]

^##^SHAPIRO et al. 2003 [121]

**Fig. 1:**
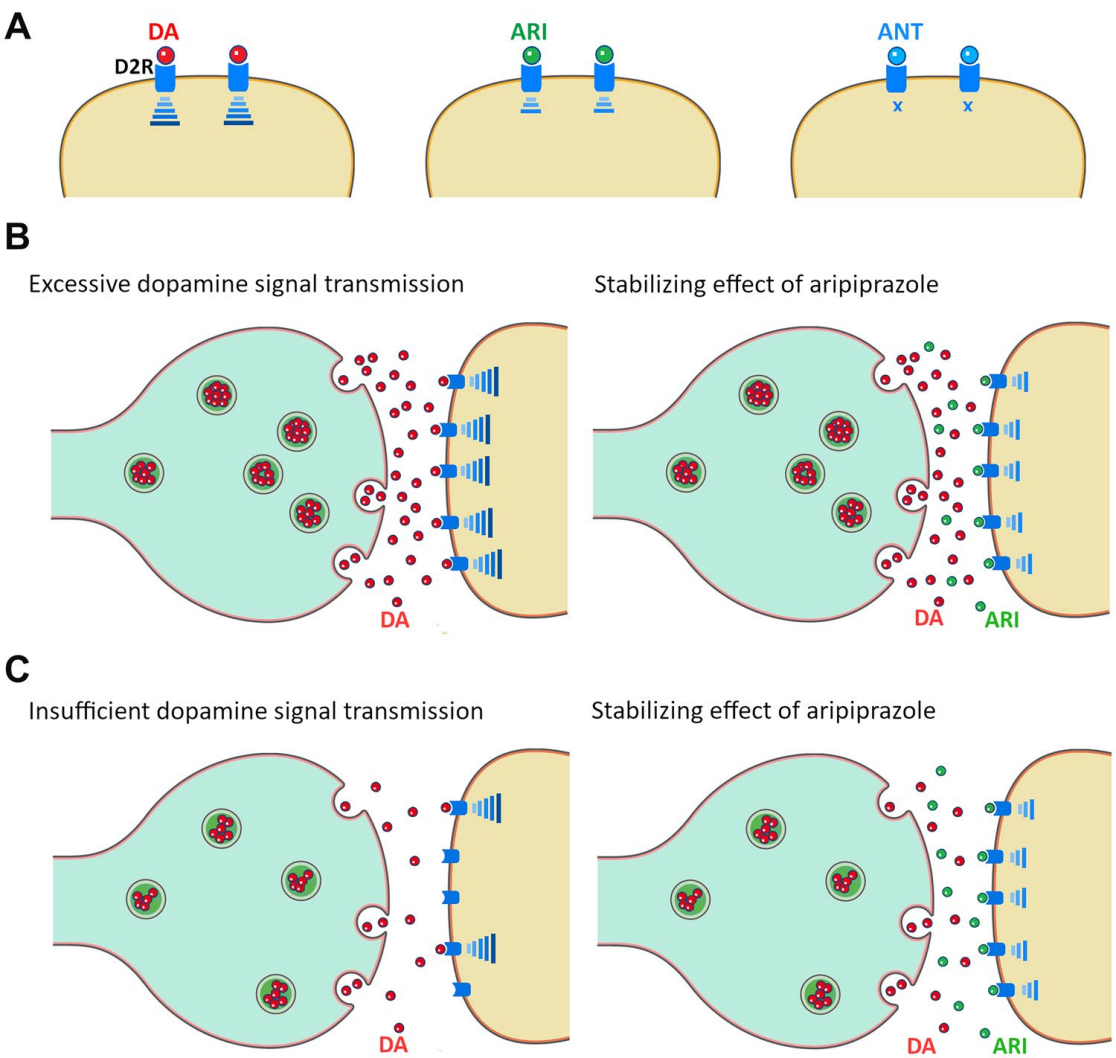
Mechanism of action of ARI. **A** ARI, as a partial agonist, reduces dopamine-mediated transmission but does not block it like an antagonist. **B** Hyperactive dopamine transmission in mesolimbic brain regions mediates positive symptoms. ARI works as a function antagonist in areas of too high dopamine levels. **C** Hypoactive dopamine transmission in mesocortical brain regions mediates negative symptoms. ARI works as a function agonist in areas of too-low dopamine levels. *DA* dopamine, *ARI* aripiprazole, *ANT* antagonist, *D2R* dopamine D2 receptor

ARI is defined as a dopamine-system stabilizer (DSS) because of its higher affinity for the D2 receptor (*K*_i_ = 0.34 nM) than for 5-HT_1A_ and 5-HT_2A_ receptors (*K*_i_ = 1.7 nM and *K*_i_ = 3.4 nM, respectively) and its stabilizing effect on dopamine (DA) neurotransmission [72]. DSS partially activates DA receptors stabilizing the balance between stimulation and blockade of DA receptors [73]. DSS blocks D2 receptors in brain regions where DA activity needs to be reduced, at the same time, it does not reduce dopamine activity in brain regions where normal DA levels are needed [74]. Indeed, a positron emission tomography (PET) performed in healthy men who received single doses of ARI (3–9 mg) has shown that ARI decreases or increases DA synthesis in individuals with high or low baseline DA levels, respectively [75]. Thus, these findings suggest that the therapeutic effects of ARI may be related to a stabilizing effect on DA synthesis capacity and dopaminergic neurotransmission. Although the ARI occupancy rate on D2 receptors needs to be greater than 90% to have to achieve a therapeutic effect, it does not produce EPS [76]. Because ARI has lower intrinsic activity than a full agonist (i.e., endogenous dopamine), therefore signal transmission is lower than that of dopamine, but not completely blocked as with an antagonist (i.e., conventional antipsychotics) (Fig. 1A).

It is postulated that the positive symptoms of schizophrenia are related to hyperactive dopamine transmission in the mesolimbic brain regions in turn, hypoactive dopamine transmission in the mesocortical system underlies the negative symptoms [77]. Due to the unique dopamine-dependent action of ARI, it helps to control both positive and negative symptoms of schizophrenia (Fig. 1B, C).

However, the unique mechanism of action is more complex possibly due to the functional selectivity of ARI. Indeed, studies indicate that ARI is a functional selective D2 ligand that exerts an effect on intracellular signaling pathways [76, 78]. An in vitro study showed that ARI caused the activation of mitogen-activated protein kinases (MAPK) and arachidonic acid pathways [78]. In addition, an in vivo functional selectivity study revealed different effects on protein kinase A (PKA), protein kinase B (Akt) and glycogen synthase kinase 3 beta (GSK3β) depending on brain regions [79]. It is worth emphasizing that elevated PKA levels in the nucleus accumbens correlated with increased expression of the GABA_A_ (β-1) receptor as well as GSK3β signaling probably modulating NMDA and GABA_A_ expression [80–82]. A study in patients with schizophrenia suggested that ARI increased GABA transmission in the prefrontal regions and this may have clinical benefits in terms of improved social competence [83]. Another study on the effects of ARI exposure on NMDA and GABA_A_ receptor binding levels revealed that ARI modulates the neurotransmission of both receptors in juvenile rats [84].

To summarize, different properties such as partial agonism and functional selectivity as well as actions at other receptor systems may be responsible for the action of ARI and the effective management of positive and negative symptoms in schizophrenia.

## Metabolism of ARI

The bioavailability of the tablet formulation of ARI is 87%, maximum plasma concentrations (*C*_max_) occur 2.8–6.8 h after drug intake (depending on the dose), and its pharmacokinetics is linear [9]. ARI is metabolized by the hepatic cytochrome P450 (CYP450) enzyme system via three biotransformation pathways: dehydrogenation, hydroxylation, and N-dealkylation [85]. The two isoenzymes, CYP2D6 and CYP3A4, are mainly involved in the metabolism and elimination of ARI. However, the CYP3A4 shows a less significant influence on the metabolism of ARI [9]. The active metabolite dehydro-aripiprazole (D-ARI) arises as a result of a dehydrogenation pathway mediated by both isoenzymes. D-ARI accounts for approximately 40% of the drug concentration in plasma [10]. Although ARI and D-ARI exhibit similar pharmacological properties, their half-lives differ significantly (ARI—75 h versus D-ARI—94 h). Several studies have shown the impact of genetic polymorphisms on the pharmacokinetic and pharmacodynamic parameters of ARI. The Food Drug Administration (FDA) and the Dutch Pharmacogenetics Working group (DPWG) recommend adjusting the dose of ARI based on the *CYP2D6* genotype. Applying a pharmacogenetic approach to ARI management can help determine a specific dosage for a patient, to ensure maximum efficacy with minimal side effects.

## Pharmacogenetics

The cytochrome P450 monooxygenases metabolize approximately 70–80% of all used drugs, including antipsychotic drugs. Their expression depends on both genetic and non-genetic factors, such as age, sex, comorbidities, and other medications [86]. The CYP450 system-mediated drug conversion can lead to detoxification, creating new, reactive molecules accelerating the process of toxic compounds elimination, and hence, general response to the therapy may differ according to the individual metabolic capacity presented by patients [87]. Thus, the overall response to therapy may vary depending on the patient's individual metabolic rate.

A recent study, performed in a population of healthy volunteers receiving a single oral dose of ARI, confirmed that the pharmacokinetic parameters are influenced by the polymorphisms of genes encoding metabolizing enzymes (*CYP2D6*, *CYP3A4*, and *CYP3A5*) and in the drug transporter (*ABCB1*) [66]. It is postulated that the pharmacodynamics of ARI can be affected by polymorphisms in dopamine D2- and serotonin-5-HT_2A_ receptors [88].

### Gene polymorphisms and drug response

#### *CYP2D6*

**Fig. 2:**
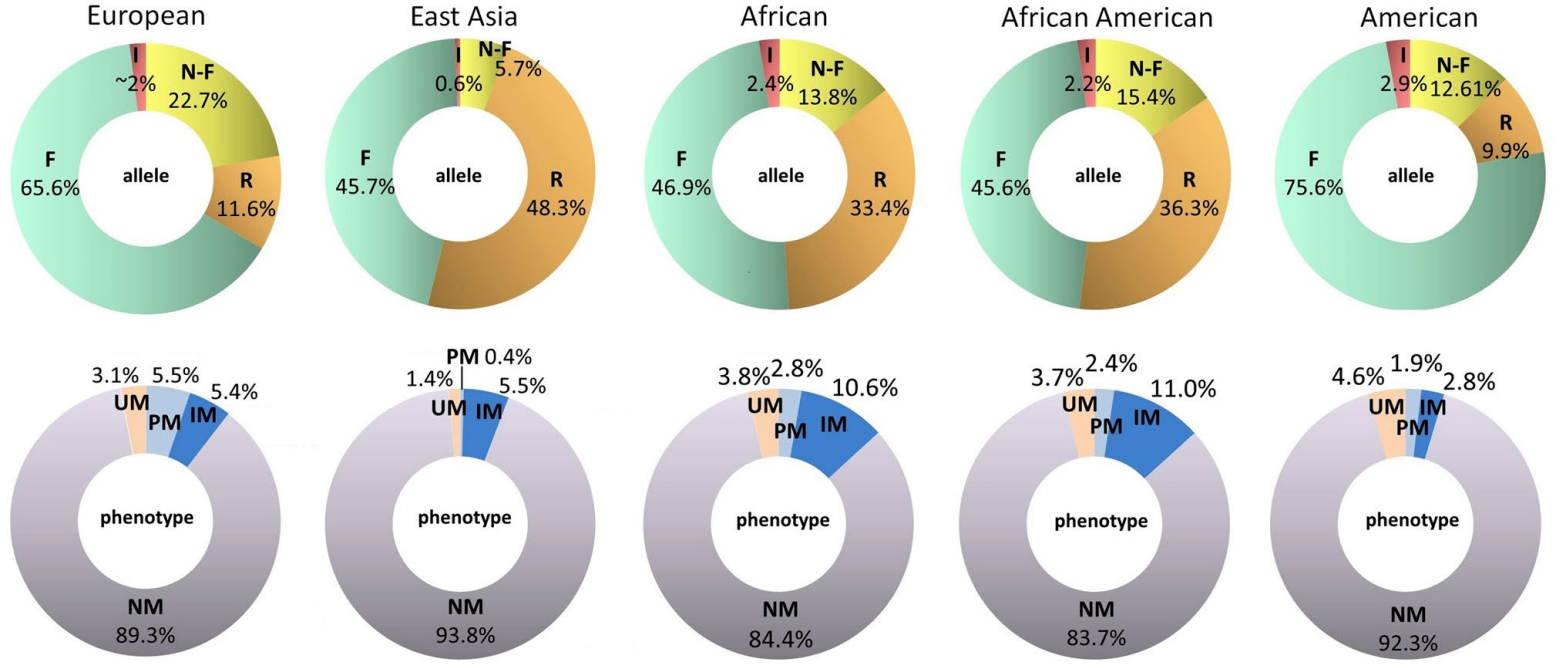
The frequencies of *CYP2D6* alleles and predicted phenotypes in the five populations. The sum of allelic frequencies is not 100% as they are average values in the given population. *I* increased functioning allele, *N-F* non-functioning allele, *R* reduced functioning allele, *F* functioning (normal) allele, *UM* ultrarapid metabolizer, *PM* poor metabolizer, *IM* intermediate metabolizer, *NM* normal metabolizer. (Diagrams have been prepared based on data provided by Gaedigk et al. 2017 [95])

Although CYP2D6 constitutes only 2% of the hepatic CYPs, it is an essential isoform involved in the metabolism of approximately 20–25% of drugs, including antidepressants, antipsychotics, β-blockers, analgesics, and tamoxifen [89]. The *CYP2D6* gene is highly polymorphic and more than 130 allelic variants have been identified so far. These variants include single nucleotide polymorphisms (SNPs), small insertions/deletions (Ins/Del) of nucleotides, deletion of the entire *CYP2D6* gene, gene duplication or multiplications as well as hybrid alleles [90, 91]. The activity of the enzyme encoded by each allele, as defined by the clinical pharmacogenomics implementation consortium (CPIC), can be either normal, reduced, or absent. The CYP2D6*1 allele is considered as a *wild-type* (so-called normal) allele that encodes enzyme with normal activity. An individual with two or one 2D6*1 alleles has a normal metabolic rate and is classified as a normal metabolizer (NM) or extensive metabolizer (EM). It is possible to predict metabolizer status based on the specific combination of alleles: ≥ 3 normal function gene copies—ultrarapid metabolizer (UM); 1 or 2 normal function alleles—normal metabolizer (NM); ≥ 2 decreased function alleles or 1 decreased function and 1 no function allele—intermediate metabolizer (IM); ≥ 2 no function alleles—poor metabolizer (PM) [92, 93]. It is well known that the frequency of *CYP2D6* alleles varies among racial and ethnic groups [94]. A study by Gaedigk and colleagues predicted phenotypes in major populations from allele frequency data [95]. The frequencies of the *CYP2D6* alleles and genetically predicted phenotypes are presented in Fig. 2.

Interestingly, the frequency of UM phenotypes is much higher in South-East compared to North-West Europe (6% in Greece and Turkey to 1% in Sweden and Denmark, except Finland—3.4%). Inversely, the frequency of loss-of-function alleles (2D6*4 and 2D6*5) was lower in Mediterranean countries and highest in Northern Europe [96].

CYP2D6 metabolizer phenotype influences the half-life of ARI, patients with PM phenotype have almost double extended mean elimination half-life (146 versus 75 h) [10], as they cannot metabolize ARI. It has been observed that when the number of active *CYP2D6* alleles decreased, AUC_0-t_ and T_*1/2*_ were higher for ARI, and AUC_0-t_ and *C*_max_ were decreased for D-ARI [66]. As recommended by the FDA and the DPWG, the standard dose should be reduced by 50% or 67% (respectively), regardless of the administration route (oral and long-acting injectable). Moreover, a quarter of the usual dose should be used in poor metabolizers (PM) taking strong inhibitors of the CYP3A4 enzyme. In addition, no action is recommended for IM or UM; however, recent studies have suggested that IM patients may require a lower dose of ARI [97, 98]. Surprisingly, a recent study in Chinese subjects has shown that *CYP2D6* rs1058164 and rs28371699 also affected the pharmacokinetics of ARI, T_1/2_, and AUC_0-∞_ but differed significantly between CYP2D6 NM and IM [99]. Due to the relatively high frequency of these SNPs in the Chinese population dose adjustment should probably be considered for IM.

#### *CYP3A4*

There are two main allelic variants of *CYP3A4*, *20 and *22, involved in the metabolism of ARI [66]. The CYP3A4*20 loss-of-function allele resulted in a higher AUC_0-t_ of ARI, and a lower AUC_0-t_ of D-ARI, thereby increasing the patient's plasma levels of ARI [66]. It seems that the CYP3A4*22 reduced functioning allele can also affect the metabolism of antipsychotics [100], but this allele did not affect the pharmacokinetics of either ARI or D-ARI [66].

#### *CYP3A5*

The role of *CYP3A5* in ARI metabolism is much less significant in comparison to *CYP2D6* [66]. However, a study has shown that the CYP3A5*3 allele may influence D-ARI/ARI ratio—lower values of this parameter were observed in individuals with genotype *3/*3 (no CYP3A5 enzyme production) compared to *1/*1 and *1/*3 genotypes [66].

#### *CYP1A2* and *UGT1A1*

Although ARI is not a substrate for CYP1A2 and UGT enzymes, a recent study suggested that polymorphism in *CYP1A2* and *UGT1A1* genes may be involved in ARI and D-ARI pharmacokinetics [101]. However, this study was performed on a small number of healthy volunteers, and thus, more studies are needed including studies in patients with schizophrenia, in order to confirm the involvement of these polymorphisms in ARI metabolism.

#### *ABCB1*

*ABCB1* gene encodes the membrane-associated protein (P-glycoprotein), a member of the superfamily of ATP-binding cassette (ABC) transporters, responsible for ATP-dependent active transport of drugs. ABCB1 protein is involved in processes, such as drug absorption, distribution, and elimination. It is postulated that the synonymous *C1236T* polymorphism influenced the expression level of the *ABCB1* gene [102, 103]; however, the results are contradictory and further studies are needed to evaluate the association between the *C1236T* polymorphism and gene expression. Interestingly, the pharmacokinetic parameters of ARI and D-ARI were influenced by the synonymous *C1236T* polymorphism in the *ABCB1* gene. The clearance of ARI, AUC_0-t,_ and C_max_ for D-ARI as well as the D-ARI/ARI ratio had higher values in *C/C* subjects compared to *T/T* subjects [66].

#### *DRD2*

There are many variants of the *DRD2* gene, including − *141 Ins/Del*, *Ser311Cys*, *C957T,* and *Taq1A*, that may affect antipsychotic response. The − *141 Ins/Del* polymorphism is a deletion of one nucleotide (cytosine) at position − 141 of the 5' promoter region. Imaging studies in healthy volunteers showed that carriers of the − *141 Del* allele have increased striatal D2 receptor density [104]. PGx testing indicated that carriers of the *Del* allele had reduced response to antipsychotic drugs [105]. The *Ser311Cys DRD2* polymorphism results in a substitution of an amino acid at position 311 (serine to cysteine). Patients with schizophrenia and the *Ser311* allele are more resistant to treatment with risperidone than patients carrying the *Cys311* allele [106]. Although the *C957T* polymorphism is a synonymous variant and does not change the amino acid sequence of the resulting protein, it can alter mRNA stability [107]. Reduced translation and mRNA stability were associated with the *T* allele [108], moreover, the *T* allele showed a protective effect against schizophrenia [109]. The *Taq1A* polymorphism is a missense variant (cytosine is replaced with thymine) resulting in an amino acid substitution at position 713 (Glu713Lys, glutamic acid to lysine). The Taq1A*1A polymorphism seems to be especially important regarding the effectiveness of antipsychotic treatments. The risk allele, *A1* (thymine) allele that reduce the expression of the *DRD2* gene decreases D2 receptor density in the striatum [104]. A further study performed on healthy volunteers, found that the *A1/A1* subjects showed increased metabolic activity in the frontal lobe compared to the *A2/A2* (*wild-type*) subjects. Thus, patients with the *A1/A1* genotype may respond better to ARI treatment [110]. Another study in patients with schizophrenia evaluated an association between the response to ARI treatment and four polymorphisms in the *DRD2* gene mentioned above [108]. The carriers of the *A1* allele with positive symptoms respond much better to ARI relative to individuals with *A2/A2* genotype. Furthermore, regarding the *C957T* polymorphism, patients with *T/T* genotype had better ARI response for excitement symptoms compared to C/C genotype. This study also revealed no association with the ARI response and two polymorphisms (*− 141 Ins/Del* and *Ser311Cys*) [108].

#### *5-HTR2A* and *5-HTR1A*

Among various polymorphisms of the *5-HTR2A* gene, the *T102C* variant is the most studied. The *C* allele decreases receptor expression and receptor binding potentials [111, 112]. A study by Lane et al. showed that patients with the *C/C* genotype respond better to risperidone treatment (especially for negative symptoms of schizophrenia) [113]. Likewise, a significantly better response to olanzapine treatment was observed in patients with positive symptoms of schizophrenia and the *C/C* genotype [114]. In contrast, another study identified that the *C* allele is associated with less effective ARI treatment on negative symptoms of schizophrenia [115]. Another polymorphism, *C1354T*, is a missense variant of the *5-HTR2A* gene resulting in an amino acid substitution at position 452 (Hys452Tyr, histidine to tyrosine). This polymorphism may alter the tertiary structure of the protein and thus may disrupt the function of the receptor. The homozygous (*His/His*) respond better to olanzapine treatment, this association was noticed in terms of positive symptoms [114].

The − *1019C/G* polymorphism in the promoter region of the *5HTR1A* gene increases its expression level both in animal models and humans. Individuals with *G/G* genotype have increased density of 5-HT_1A_ receptor density in presynaptic raphe neurons [116]. In patients with the *C/C* genotype olanzapine or perospirone more effectively improved the cognitive deficit of schizophrenia (attention) than in patients having *G/C* and *G/G* genotypes [116]. Previous studies showed that the response to treatment with various antipsychotics was limited when the *G* allele was present, possibly due to the increased receptor density, which may result in the lower efficacy of antipsychotic drugs [117, 118].

### Gene polymorphisms and metabolic side effects

Generally, ARI is well tolerated and not associated with significant EPS or raised prolactin concentrations. However, in some patients, it can cause side effects, such as increased blood glucose or cholesterol levels. Typically, ARI leads to low prolactin elevation, but in less than 5% of patients can sometimes cause hyperprolactinemia [119]. A recent study performed on healthy volunteers revealed that polymorphisms in specific genes can affect the levels of prolactin, C-peptide, insulin, and cholesterol [120]. Table 3 presents the relationship between metabolic parameters and gene polymorphisms identified after 5 days of ARI administration.

**Table 3:** Metabolic effects of aripiprazole in healthy volunteers

Metabolic parameters	Gene	Polymorphisms/phenotype	Effects
Prolactin	*CYP3A4*	PM	↑ Concentrations compared to IM and NM phenotypes
	*ABCB1*	rs10280101 rs12720067 rs11983225	↑ Concentrations in subjects with *A*-*C*-*T* haplotype
C-peptide	*COMT*	rs4680	↑ Levels in *G/G* genotype
		rs13306278	↑ Levels in *T* allele carriers
Insulin	*BDNF*	rs6265	Greater increment in *C/C* genotype
Cholesterol	*HTR2A*	rs6314	↑ Concentrations in *C/C* genotype

The table is based on information from a study in healthy volunteers [120]

## Conclusions

In this review, we discuss the possible association between gene polymorphisms and ARI response. Although the FDA and DPWG recommended dosage adjustments for patients who are *CYP2D6* poor metabolizers, it seems that other genetic variations are also related to pharmacokinetic, pharmacodynamics, and side effects of the drug. The specific genetic profile of a patient can determine the effectiveness and tolerability of ARI. We believe that more targeted pharmacogenetics testing prior to prescribing ARI will provide the opportunity for personalized medicine to treat schizophrenia, thereby improving clinical outcomes and patient satisfaction. However, extensive pharmacogenetic studies are needed to assess the relevance of specific gene polymorphisms in response to the drug, which will be included in future diagnostic panels.

## Data Availability

Not applicable.

## References

[1] Mailman RB, Murthy V (2010). Third generation antipsychotic drugs: partial agonism or receptor functional selectivity?. Curr Pharm Des.

[2] Orsolini L, De Berardis D, Volpe U (2020). Up-to-date expert opinion on the safety of recently developed antipsychotics. Expert Opin Drug Saf.

[3] Li P, Snyder GL, Vanover KE (2016). Dopamine targeting drugs for the treatment of schizophrenia: past, present and future. Curr Top Med Chem.

[4] Smith S, Wheeler MJ, Murray R, O'Keane V (2002). The effects of antipsychotic-induced hyperprolactinaemia on the hypothalamic-pituitary-gonadal axis. J Clin Psychopharmacol.

[5] Pollmächer T, Wright JA (2015). Treatment of schizophrenia. International Encyclopedia of the Social and Behavioral Sciences.

[6] Endomba FT, Tankeu AT, Nkeck JR, Tochie JN (2020). Leptin and psychiatric illnesses: does leptin play a role in antipsychotic-induced weight gain?. Lipids Health Dis.

[7] Uçok A, Gaebel W (2008). Side effects of atypical antipsychotics: a brief overview. World Psychiatry.

[8] Kondej M, Stępnicki P, Kaczor AA (2018). Multi-target approach for drug discovery against schizophrenia. Int J Mol Sci.

[9] Soria-Chacartegui P, Villapalos-García G, Zubiaur P, Abad-Santos F, Koller D (2021). Genetic polymorphisms associated with the pharmacokinetics, pharmacodynamics and adverse effects of olanzapine, aripiprazole and risperidone. Front Pharmacol.

[10] Kneller LA, Zubiaur P, Koller D, Abad-Santos F, Hempel G (2021). Influence of CYP2D6 phenotypes on the pharmacokinetics of aripiprazole and dehydro-aripiprazole using a physiologically based pharmacokinetic approach. Clin Pharmacokinet.

[11] Baandrup L (2020). Polypharmacy in schizophrenia. Basic Clin Pharmacol Toxicol.

[12] Lally J, MacCabe JH (2015). Antipsychotic medication in schizophrenia: a review. Br Med Bull.

[13] Kim DD, Barr AM, Lian L, Yuen JWY, Fredrikson D, Honer WG (2021). Efficacy and tolerability of aripiprazole versus D. NPJ Schizophr.

[14] Han M, Huang XF, Deng C (2009). Aripiprazole differentially affects mesolimbic and nigrostriatal dopaminergic transmission: implications for long-term drug efficacy and low extrapyramidal side-effects. Int J Neuropsychopharmacol.

[15] Gettu N, Saadabadi A. Aripiprazole. [Updated 2022 May 21]. In: StatPearls [Internet]. Treasure Island (FL): StatPearls Publishing; 2022 Jan-. PMID: 31613519. Available from: https://www.ncbi.nlm.nih.gov/books/NBK547739/

[16] Lee HJ, Ormandy CJ (2012). Interplay between progesterone and prolactin in mammary development and implications for breast cancer. Mol Cell Endocrinol.

[17] Wang M, Wu X, Chai F, Zhang Y, Jiang J (2016). Plasma prolactin and breast cancer risk: a meta- analysis. Sci Rep.

[18] Taipale H, Solmi M, Lähteenvuo M, Tanskanen A, Correll CU, Tiihonen J (2021). Antipsychotic use and risk of breast cancer in women with schizophrenia: a nationwide nested case-control study in Finland. Lancet Psychiatry.

[19] Taylor DM (2003). Aripiprazole: a review of its pharmacology and clinical use. Int J Clin Pract.

[20] Plows JF, Stanley JL, Baker PN, Reynolds CM, Vickers MH (2018). The pathophysiology of gestational diabetes mellitus. Int J Mol Sci.

[21] Damkier P, Videbech P (2018). The safety of second-generation antipsychotics during pregnancy: a clinically focused review. CNS Drugs.

[22] Galbally M, Frayne J, Watson SJ, Snellen M (2018). Aripiprazole and pregnancy: a retrospective, multicentre study. J Affect Disord.

[23] Fernández-Abascal B, Recio-Barbero M, Sáenz-Herrero M, Segarra R (2021). Long-acting injectable aripiprazole in pregnant women with schizophrenia: a case-series report. Ther Adv Psychopharmacol.

[24] Lee BH, Kim YK, Park SH (2006). Using aripiprazole to resolve antipsychotic-induced symptomatic hyperprolactinemia: a pilot study. Prog Neuropsychopharmacol Biol Psychiatry.

[25] Sheldrick AJ, Gründer G (2008). Aripiprazole reduces serum prolactin in a woman with prolactinoma and acute psychosis. Pharmacopsychiatry.

[26] Muench J, Hamer AM (2010). Adverse effects of antipsychotic medications. Am Fam Physician.

[27] Adams CE, Rathbone J, Thornley B, Clarke M, Borrill J, Wahlbeck K (2005). Chlorpromazine for schizophrenia: a Cochrane systematic review of 50 years of randomised controlled trials. BMC Med.

[28] Liu X, De Haan S (2009). Chlorpromazine dose for people with schizophrenia. Cochrane Database Syst Rev.

[29] Solmi M, Murru A, Pacchiarotti I, Undurraga J, Veronese N, Fornaro M (2017). Safety, tolerability, and risks associated with first- and second-generation antipsychotics: a state-of-the-art clinical review. Ther Clin Risk Manag.

[30] Wenzel-Seifert K, Wittmann M, Haen E (2011). QTc prolongation by psychotropic drugs and the risk of Torsade de Pointes. Dtsch Arztebl Int.

[31] Pacher P, Kecskemeti V (2004). Cardiovascular side effects of new antidepressants and antipsychotics: new drugs, old concerns?. Curr Pharm Des.

[32] Hennessy S, Bilker WB, Knauss JS, Kimmel SE, Margolis DJ, Morrison MF (2004). Comparative cardiac safety of low-dose thioridazine and low-dose haloperidol. Br J Clin Pharmacol.

[33] Feinberg SM, Fariba KA, Saadabadi A. Thioridazine. [Updated 2022 May 2]. In: StatPearls [Internet]. Treasure Island (FL): StatPearls Publishing; 2022 Jan-. Available from: https://www.ncbi.nlm.nih.gov/books/NBK459140/

[34] Kudo S, Ishizaki T (1999). Pharmacokinetics of haloperidol: an update. Clin Pharmacokinet.

[35] Girard TD, Exline MC, Carson SS, Hough CL, Rock P, Gong MN (2018). Haloperidol and ziprasidone for treatment of delirium in critical illness. N Engl J Med.

[36] Rahman S, Marwaha R. Haloperidol. 2022 Jul 4. In: StatPearls [Internet]. Treasure Island (FL): StatPearls Publishing; 2022 Jan–. PMID: 32809727.

[37] Huhn M, Nikolakopoulou A, Schneider-Thoma J, Krause M, Samara M, Peter N (2019). Comparative efficacy and tolerability of 32 oral antipsychotics for the acute treatment of adults with multi-episode schizophrenia: a systematic review and network meta-analysis. Lancet.

[38] Pinder RM, Brogden RN, Swayer R, Speight TM, Spencer R, Avery GS (1976). Pimozide: a review of its pharmacological properties and therapeutic uses in psychiatry. Drugs.

[39] Tueth MJ, Cheong JA (1993). Clinical uses of pimozide. South Med J.

[40] Vardanyan R, Vardanyan R (2017). Piperidine-Based Nonfused Biheterocycles With C-N and C–C Coupling. Piperidine-Based Drug Discovery.

[41] Gardner DM, Baldessarini RJ, Waraich P (2005). Modern antipsychotic drugs: a critical overview. CMAJ.

[42] Siragusa S, Bistas KG, Saadabadi A. Fluphenazine. [Updated 2022 May 8]. In: StatPearls [Internet]. Treasure Island (FL): StatPearls Publishing; 2022 Jan-. PMID: 29083807.Available from: https://www.ncbi.nlm.nih.gov/books/NBK459194/29083807

[43] Aronson JK, Aronson JK (2015). Fluphenazine. Meyler's side effects of drugs. The International Encyclopedia of adverse drug reactions and interactions.

[44] Praharaj SK, Jana AK, Goyal N, Sinha VK (2011). Metformin for olanzapine-induced weight gain: a systematic review and meta-analysis. Br J Clin Pharmacol.

[45] Carli M, Kolachalam S, Longoni B, Pintaudi A, Baldini M, Aringhieri S (2021). Atypical antipsychotics and metabolic syndrome: from molecular mechanisms to clinical differences. Pharmaceuticals (Basel).

[46] Lieberman JA, Stroup TS, McEvoy JP, Swartz MS, Rosenheck RA, Perkins DO (2005). Effectiveness of antipsychotic drugs in patients with chronic schizophrenia. N Engl J Med.

[47] Dayabandara M, Hanwella R, Ratnatunga S, Seneviratne S, Suraweera C, de Silva VA (2017). Antipsychotic-associated weight gain: management strategies and impact on treatment adherence. Neuropsychiatr Dis Treat.

[48] Thomas K, Saadabadi A. Olanzapine. [Updated 2022 Sep 8]. In: StatPearls [Internet]. Treasure Island (FL): StatPearls Publishing; 2022 Jan-. PMID: 30422498. Available from: https://www.ncbi.nlm.nih.gov/books/NBK532903/

[49] Jahołkowski P, Mierzejewski P, Świtaj P (2019). Clozapine-induced myocarditis. Adv Psychiatry Neurol/Postępy Psychiatrii i Neurologii.

[50] Montejo AL (2008). Prolactin awareness: an essential consideration for physical health in schizophrenia. Eur Neuropsychopharmacol.

[51] Fitton A, Heel RC (1990). Clozapine. A review of its pharmacological properties, and therapeutic use in schizophrenia. Drugs.

[52] Miller DD (2000). Review and management of clozapine side effects. J Clin Psychiatry.

[53] Blackman G, Lisshammar JEL, Zafar R, Pollak TA, Pritchard M, Cullen AE (2021). Clozapine response in schizophrenia and hematological changes. J Clin Psychopharmacol.

[54] Haidary HA, Padhy RK. Clozapine. [Updated 2021 Dec 6]. In: StatPearls [Internet]. Treasure Island (FL): StatPearls Publishing; 2022 Jan-. PMID: 30571020. Available from: https://www.ncbi.nlm.nih.gov/books/NBK535399/

[55] Rummel-Kluge C, Komossa K, Schwarz S, Hunger H, Schmid F, Lobos CA (2010). Head-to-head comparisons of metabolic side effects of second generation antipsychotics in the treatment of schizophrenia: a systematic review and meta-analysis. Schizophr Res.

[56] McNeil SE, Gibbons JR, Cogburn M. Risperidone. [Updated 2022 May 17]. In: StatPearls [Internet]. Treasure Island (FL): StatPearls Publishing; 2022 Jan-. PMID: 29083663. Available from: https://www.ncbi.nlm.nih.gov/books/NBK459313/29083663

[57] DeVane CL, Nemeroff CB (2001). Clinical pharmacokinetics of quetiapine: an atypical antipsychotic. Clin Pharmacokinet.

[58] Sanford M, Keating GM (2012). Quetiapine: a review of its use in the management of bipolar depression. CNS Drugs.

[59] Conley RR, Kelly DL (2005). Second-generation antipsychotics for schizophrenia: a review of clinical pharmacology and medication-associated side effects. Isr J Psychiatry Relat Sci.

[60] Maan JS, Ershadi M, Khan I, et al. Quetiapine. [Updated 2022 Sep 2]. In: StatPearls [Internet]. Treasure Island (FL): StatPearls Publishing; 2022 Jan-. PMID: 29083706. Available from: https://www.ncbi.nlm.nih.gov/books/NBK459145/

[61] Greenberg WM, Citrome L (2007). Ziprasidone for schizophrenia and bipolar disorder: a review of the clinical trials. CNS Drug Rev.

[62] Swainston Harrison T, Scott LJ (2006). Ziprasidone: a review of its use in schizophrenia and schizoaffective disorder. CNS Drugs.

[63] Bouchette D, Fariba KA, Marwaha R. Ziprasidone. [Updated 2022 May 8]. In: StatPearls [Internet]. Treasure Island (FL): StatPearls Publishing; 2022 Jan-. PMID: 28846230. Available from: https://www.ncbi.nlm.nih.gov/books/NBK448157/

[64] Potkin SG, Saha AR, Kujawa MJ, Carson WH, Ali M, Stock E (2003). Aripiprazole, an antipsychotic with a novel mechanism of action, and risperidone vs placebo in patients with schizophrenia and schizoaffective disorder. Arch Gen Psychiatry.

[65] Dean L, Kane M, Pratt VM, Scott SA, Pirmohamed M, Esquivel B, Kane MS, Kattman BL, Malheiro AJ (2012). Clozapine therapy and CYP genotype. Medical genetics summaries.

[66] Belmonte C, Ochoa D, Román M, Saiz-Rodríguez M, Wojnicz A, Gómez-Sánchez CI (2018). Influence of CYP2D6, CYP3A4, CYP3A5 and ABCB1 polymorphisms on pharmacokinetics and safety of aripiprazole in healthy volunteers. Basic Clin Pharmacol Toxicol.

[67] Corponi F, Fabbri C, Bitter I, Montgomery S, Vieta E, Kasper S (2019). Novel antipsychotics specificity profile: a clinically oriented review of lurasidone, brexpiprazole, cariprazine and lumateperone. Eur Neuropsychopharmacol.

[68] Stahl SM (2016). Mechanism of action of brexpiprazole: comparison with aripiprazole. CNS Spectr.

[69] Goff DC (2015). Brexpiprazole: a new antipsychotic following in the footsteps of aripiprazole. Am J Psychiatry.

[70] Stahl SM (2016). Mechanism of action of cariprazine. CNS Spectr.

[71] Kikuchi T, Maeda K, Suzuki M, Hirose T, Futamura T, McQuade RD (2021). Discovery research and development history of the dopamine D. Neuropsychopharmacol Rep.

[72] Mamo D, Graff A, Mizrahi R, Shammi CM, Romeyer F, Kapur S (2007). Differential effects of aripiprazole on D(2), 5-HT(2), and 5-HT(1A) receptor occupancy in patients with schizophrenia: a triple tracer PET study. Am J Psychiatry.

[73] Stahl SM (2001). Dopamine system stabilizers, aripiprazole, and the next generation of antipsychotics, part 2: illustrating their mechanism of action. J Clin Psychiatry.

[74] Stahl SM (2001). Dopamine system stabilizers, aripiprazole, and the next generation of antipsychotics, part 1, "Goldilocks" actions at dopamine receptors. J Clin Psychiatry.

[75] Ito H, Takano H, Arakawa R, Takahashi H, Kodaka F, Takahata K (2012). Effects of dopamine D2 receptor partial agonist antipsychotic aripiprazole on dopamine synthesis in human brain measured by PET with L-[β-11C]DOPA. PLoS ONE.

[76] Tuplin EW, Holahan MR (2017). Aripiprazole, a drug that displays partial agonism and functional selectivity. Curr Neuropharmacol.

[77] Brisch R, Saniotis A, Wolf R, Bielau H, Bernstein HG, Steiner J (2014). The role of dopamine in schizophrenia from a neurobiological and evolutionary perspective: old fashioned, but still in vogue. Front Psychiatry.

[78] Urban JD, Vargas GA, von Zastrow M, Mailman RB (2007). Aripiprazole has functionally selective actions at dopamine D2 receptor-mediated signaling pathways. Neuropsychopharmacology.

[79] Pan B, Chen J, Lian J, Huang XF, Deng C (2015). Unique effects of acute aripiprazole treatment on the dopamine D2 receptor downstream cAMP-PKA and Akt-GSK3β signalling pathways in rats. PLoS ONE.

[80] Pan B, Lian J, Huang XF, Deng C (2016). Aripiprazole increases the PKA signalling and expression of the GABAA receptor and CREB1 in the nucleus accumbens of rats. J Mol Neurosci.

[81] Pan B, Huang XF, Deng C (2016). Chronic administration of aripiprazole activates GSK3β-dependent signalling pathways, and up-regulates GABAA receptor expression and CREB1 activity in rats. Sci Rep.

[82] Pan B, Lian J, Deng C (2018). Chronic antipsychotic treatment differentially modulates protein kinase A- and glycogen synthase kinase 3 beta-dependent signaling pathways, N-methyl-D-aspartate receptor and γ-aminobutyric acid A receptors in nucleus accumbens of juvenile rats. J Psychopharmacol.

[83] Lee JS, Lee JD, Park HJ, Oh MK, Chun JW, Kim SJ (2013). Is the GABA system related to the social competence improvement effect of aripiprazole? An (18)F-Fluoroflumazenil PET study. Psychiatry Investig.

[84] Lian J, Deng C (2019). Early antipsychotic exposure affects NMDA and GABAA receptor binding in the brains of juvenile rats. Psychiatry Res.

[85] McGavin JK, Goa KL (2002). Aripiprazole. CNS Drugs.

[86] Zanger UM, Schwab M (2013). Cytochrome P450 enzymes in drug metabolism: regulation of gene expression, enzyme activities, and impact of genetic variation. Pharmacol Ther.

[87] Zhao M, Ma J, Li M, Zhang Y, Jiang B, Zhao X (2021). Cytochrome P450 enzymes and drug metabolism in humans. Int J Mol Sci.

[88] Blasi G, Selvaggi P, Fazio L, Antonucci LA, Taurisano P, Masellis R (2015). Variation in dopamine D2 and serotonin 5-HT2A receptor genes is associated with working memory processing and response to treatment with antipsychotics. Neuropsychopharmacology.

[89] Taylor C, Crosby I, Yip V, Maguire P, Pirmohamed M, Turner RM (2020). A review of the important role of CYP2D6 in pharmacogenomics. Genes.

[90] Del Tredici AL, Malhotra A, Dedek M, Espin F, Roach D, Zhu GD (2018). Frequency of CYP2D6 alleles including structural variants in the United States. Front Pharmacol.

[91] Kane M. CYP2D6 Overview: Allele and Phenotype Frequencies. 2021 Oct 15. In: Pratt VM, Scott SA, Pirmohamed M, et al., (eds). Medical Genetics Summaries [Internet]. Bethesda (MD): National Center for Biotechnology Information (US); 2012-. Available from: https://www.ncbi.nlm.nih.gov/books/NBK574601/28520340

[92] Owen RP, Sangkuhl K, Klein TE, Altman RB (2009). Cytochrome P450 2D6. Pharmacogenet Genomics.

[93] Zhang JP, Malhotra AK (2011). Pharmacogenetics and antipsychotics: therapeutic efficacy and side effects prediction. Expert Opin Drug Metab Toxicol.

[94] Bradford LD (2002). CYP2D6 allele frequency in European Caucasians, Asians, Africans and their descendants. Pharmacogenomics.

[95] Gaedigk A, Sangkuhl K, Whirl-Carrillo M, Klein T, Leeder JS (2017). Prediction of CYP2D6 phenotype from genotype across world populations. Genet Med.

[96] Petrović J, Pešić V, Lauschke VM (2020). Frequencies of clinically important CYP2C19 and CYP2D6 alleles are graded across Europe. Eur J Hum Genet.

[97] Tveito M, Molden E, Høiseth G, Correll CU, Smith RL (2020). Impact of age and CYP2D6 genetics on exposure of aripiprazole and dehydroaripiprazole in patients using long-acting injectable versus oral formulation: relevance of poor and intermediate metabolizer status. Eur J Clin Pharmacol.

[98] Zhang X, Xiang Q, Zhao X, Ma L, Cui Y (2019). Association between aripiprazole pharmacokinetics and CYP2D6 phenotypes: a systematic review and meta-analysis. J Clin Pharm Ther.

[99] Zhang X, Liu C, Zhou S, Xie R, He X, Wang Z (2021). Influence of YP2D6 gene polymorphisms on the pharmacokinetics of aripiprazole in healthy Chinese subjects. Pharmacogenomics.

[100] van der Weide K, van der Weide J (2014). The influence of the CYP3A4*22 polymorphism on serum concentration of quetiapine in psychiatric patients. J Clin Psychopharmacol.

[101] Koller D, Saiz-Rodríguez M, Zubiaur P, Ochoa D, Almenara S, Román M (2020). The effects of aripiprazole and olanzapine on pupillary light reflex and its relationship with pharmacogenetics in a randomized multiple-dose trial. Br J Clin Pharmacol.

[102] Illmer T, Schuler US, Thiede C, Schwarz UI, Kim RB, Gotthard S (2002). MDR1 gene polymorphisms affect therapy outcome in acute myeloid leukemia patients. Cancer Res.

[103] Hemauer SJ, Nanovskaya TN, Abdel-Rahman SZ, Patrikeeva SL, Hankins GD, Ahmed MS (2010). Modulation of human placental P-glycoprotein expression and activity by MDR1 gene polymorphisms. Biochem Pharmacol.

[104] Jönsson EG, Nöthen MM, Grünhage F, Farde L, Nakashima Y, Propping P (1999). Polymorphisms in the dopamine D2 receptor gene and their relationships to striatal dopamine receptor density of healthy volunteers. Mol Psychiatry.

[105] Wu S, Xing Q, Gao R, Li X, Gu N, Feng G (2005). Response to chlorpromazine treatment may be associated with polymorphisms of the DRD2 gene in Chinese schizophrenic patients. Neurosci Lett.

[106] Lane HY, Lee CC, Chang YC, Lu CT, Huang CH, Chang WH (2004). Effects of dopamine D2 receptor Ser311Cys polymorphism and clinical factors on risperidone efficacy for positive and negative symptoms and social function. Int J Neuropsychopharmacol.

[107] Duan J, Wainwright MS, Comeron JM, Saitou N, Sanders AR, Gelernter J (2003). Synonymous mutations in the human dopamine receptor D2 (DRD2) affect mRNA stability and synthesis of the receptor. Hum Mol Genet.

[108] Shen YC, Chen SF, Chen CH, Lin CC, Chen SJ, Chen YJ (2009). Effects of DRD2/ANKK1 gene variations and clinical factors on aripiprazole efficacy in schizophrenic patients. J Psychiatr Res.

[109] González-Castro TB, Hernández-Díaz Y, Juárez-Rojop IE, López-Narváez ML, Tovilla-Zárate CA, Genis-Mendoza A (2016). The role of C957T, TaqI and Ser311Cys polymorphisms of the DRD2 gene in schizophrenia: systematic review and meta-analysis. Behav Brain Funct.

[110] Kwon JS, Kim E, Kang DH, Choi JS, Yu KS, Jang IJ (2008). Taq1A polymorphism in the dopamine D2 receptor gene as a predictor of clinical response to aripiprazole. Eur Neuropsychopharmacol.

[111] Polesskaya OO, Sokolov BP (2002). Differential expression of the "C" and "T" alleles of the 5-HT2A receptor gene in the temporal cortex of normal individuals and schizophrenics. J Neurosci Res.

[112] Turecki G, Brière R, Dewar K, Antonetti T, Lesage AD, Séguin M (1999). Prediction of level of serotonin 2A receptor binding by serotonin receptor 2A genetic variation in postmortem brain samples from subjects who did or did not commit suicide. Am J Psychiatry.

[113] Lane HY, Chang YC, Chiu CC, Chen ML, Hsieh MH, Chang WH (2002). Association of risperidone treatment response with a polymorphism in the 5-HT(2A) receptor gene. Am J Psychiatry.

[114] Olajossy-Hilkesberger L, Godlewska B, Schosser-Haupt A, Olajossy M, Wojcierowski J, Landowski J (2011). Polymorphisms of the 5-HT2A receptor gene and clinical response to olanzapine in paranoid schizophrenia. Neuropsychobiology.

[115] Chen SF, Shen YC, Chen CH (2009). HTR2A A-1438G/T102C polymorphisms predict negative symptoms performance upon aripiprazole treatment in schizophrenic patients. Psychopharmacology.

[116] Sumiyoshi T, Tsunoda M, Higuchi Y, Itoh T, Seo T, Itoh H (2010). Serotonin-1A receptor gene polymorphism and the ability of antipsychotic drugs to improve attention in schizophrenia. Adv Ther.

[117] Reynolds GP, Arranz B, Templeman LA, Fertuzinhos S, San L (2006). Effect of 5-HT1A receptor gene polymorphism on negative and depressive symptom response to antipsychotic treatment of drug-naive psychotic patients. Am J Psychiatry.

[118] Wang L, Fang C, Zhang A, Du J, Yu L, Ma J (2008). The –1019 C/G polymorphism of the 5-HT(1)A receptor gene is associated with negative symptom response to risperidone treatment in schizophrenia patients. J Psychopharmacol.

[119] Kane JM, Meltzer HY, Carson WH, McQuade RD, Marcus RN, Sanchez R (2007). Aripiprazole for treatment-resistant schizophrenia: results of a multicenter, randomized, double-blind, comparison study versus perphenazine. J Clin Psychiatry.

[120] Koller D, Almenara S, Mejía G, Saiz-Rodríguez M, Zubiaur P, Román M (2021). Metabolic effects of aripiprazole and olanzapine multiple-dose treatment in a randomised crossover clinical trial in healthy volunteers: association with pharmacogenetics. Adv Ther.

[121] Shapiro DA, Renock S, Arrington E, Chiodo LA, Liu LX, Sibley DR (2003). Aripiprazole, a novel atypical antipsychotic drug with a unique and robust pharmacology. Neuropsychopharmacology.

